# Modeling Dropout Intentions in Sports Teams: Coach–Athlete and Peer Motivational Interdependence

**DOI:** 10.3390/bs16091589

**Published:** 2026-09-07

**Authors:** Tom De Clerck, Leen Haerens, Arne Bouten, Elisa Lefever, Tom Loeys

**Affiliations:** 1 Department of Movement and Sport Sciences, Ghent University, B-9000 Ghent, Belgium; tom.declerck@ugent.be (T.D.C.); leen.haerens@ugent.be (L.H.); arne.bouten@ugent.be (A.B.); elisa.lefever@ugent.be (E.L.); 2 Department of Data-Analysis, Faculty of Psychology and Educational Sciences, Ghent University, B-9000 Ghent, Belgium

**Keywords:** Actor–Partner Interdependence Model, Group Actor–Partner Interdependence Model, motivation, peer dynamics, Self-Determination Theory

## Abstract

(1) Background: Sports participation offers vital physical, psychological, and social benefits, yet dropout rates remain high. Drawing on Self-Determination Theory, previous research has primarily analyzed dropout from an individual-centered perspective. This study extends the literature by examining how the motivational orientations of coaches and peers relate to athletes’ dropout intentions; (2) Method: Data were collected from 268 athletes and 50 coaches in Flanders (Belgium). Coach–athlete interdependencies were analyzed using the Actor–Partner Interdependence Model for one-with-many designs, while peer dynamics were assessed using the Group Actor–Partner Interdependence Model; (3) Results: Analyses of coach–athlete interdependencies revealed robust individual actor effects but no partner effects. Analyses of peer dynamics indicated that dropout intentions were also related to the combination of athletes’ own and their peers’ motivation. Athletes reported higher dropout intentions when their identified regulation was low relative to that of their peers. Amotivation displayed a paradoxical pattern: athletes’ own amotivation was associated with higher dropout intentions, whereas higher peer amotivation, and a smaller discrepancy between an athlete’s own and their peers’ amotivation, was associated with lower dropout intentions. These exploratory findings require replication; (4) Conclusions: Integrating analyses of coach–athlete and peer motivational interdependence highlights the importance of considering the broader team motivational context when addressing intentions to drop out of sport.

## 1. Introduction

Sports club participation is recognized for its potential to foster long-term physical, social, and psychological health (Doré et al., 2019; Palomaeki et al., 2018). However, despite these benefits, research points to concerning rates of sports dropout, often beginning around the age of 12 and persisting well into adulthood (Eime et al., 2019). This trend has stimulated a substantial body of research aimed at understanding why athletes disengage. Among the theoretical lenses applied, Self-Determination Theory (SDT; Deci & Ryan, 2000; Ryan & Deci, 2024) has emerged as one of the most influential, positing that the extent to which athletes’ motivation is self-determined plays a central role in predicting persistence or withdrawal. Specifically, more self-determined forms of motivation (i.e., intrinsic motivation and identified regulation) have been associated with lower intentions to dropout, whereas less self-determined forms (i.e., introjected and external regulation, as well as amotivation) have been linked to higher dropout intentions (Fabra et al., 2023; Saward et al., 2024; J. H. Yang et al., 2025).

Although SDT research has generated valuable insights, it has traditionally adopted an individual-centered perspective, emphasizing athletes’ personal motivations as the primary drivers of their own dropout intentions (e.g., Jowett & Nezlek, 2012; Liu et al., 2025). This focus implicitly assumes that the motivations underlying dropout can be understood independently of athletes’ social context. Yet, many athletes within sport clubs continuously interact with key social agents, including coaches and peers. Consequently, scholars increasingly argue that the personal characteristics of coaches and peers (including their motivational orientations) also may shape athlete outcomes, such as dropout intentions (Fonteyn et al., 2022; Liu et al., 2025). In turn, athletes’ personal characteristics may likewise influence coaches and peers.

However, empirical evidence for such mutual influences remains limited. Most existing studies adopt a unidirectional perspective in which the characteristics of one actor (e.g., a coach’s motivation) are linked primarily to that actor’s own outcomes (e.g., the coach’s intentions to discontinue involvement; De Clerck et al., 2024). The few studies that have examined reciprocal influences in sport have mainly focused on the dyadic coach–athlete relationship. Drawing on the Actor–Partner Interdependence Model (APIM; Kenny et al., 2006), this line of research has demonstrated that coaches’ and athletes’ characteristics, including their motivation, can mutually influence one another within close one-to-one relationships (e.g., Fonteyn et al., 2022; Jowett et al., 2013; Stebbings et al., 2016; S. X. Yang et al., 2015). However, this strong focus on dyadic interactions limits the generalizability of these findings to typical sport club settings. In both team and individual sports, athletes are usually part of larger groups that interact with the same coach, rendering a purely one-on-one perspective insufficient. Moreover, within such team contexts, the potential role of peers in shaping motivational processes remains largely underexplored (Fonteyn et al., 2022).

This study fills these gaps, examining the role of the broader motivational context of sport teams—including both coaches and peers—in relation to athletes’ dropout intentions. For this purpose, we rely on two innovative approaches. First, we extend the Actor–Partner Interdependence Model (APIM) to multi-member sport teams by applying a one-with-many design (APIM-OWM), in which a single coach is part of a team with multiple athletes. This model examines whether coaches’ and athletes’ motivational regulations are associated with both their own intentions to discontinue involvement and those of the other party. Second, and as a key contribution, we move beyond APIM, applying the Group Actor–Partner Interdependence Model (GAPIM; Kenny & Garcia, 2012) to investigate how the team’s motivational landscape relates to athletes’ dropout intentions. Specifically, this approach disentangles the effects of athletes’ own motivation, their teammates’ motivation, and the correspondence between the two. By employing an extended APIM-OWM and GAPIM, this exploratory study examines motivational dynamics within sport teams, thereby advancing understanding of how broader team environments may contribute to dropout intentions.

### 1.1. SDT’s Quality of Motivation and Dropout

SDT distinguishes qualitative forms of motivation according to the degree to which they are self-determined or volitional in nature (Deci & Ryan, 2000; Ryan & Deci, 2024). These range from intrinsic motivation (engaging in an activity out of interest and enjoyment), through identified regulation (participating because the activity is perceived as personally valuable), introjected regulation (behavior driven by internal pressures such as guilt or pride), and external regulation (participation in response to external pressures such as approval or punishment), to amotivation (a lack or very low intensity of motivation). The more volitional regulations (intrinsic, identified) are referred to as autonomous motivation, while the more pressured forms (introjected, external) are referred to as controlled motivation.

SDT research has consistently highlighted the central role of motivation in predicting discontinuance across a broad range of populations including coaches (De Clerck et al., 2024), volunteers (Forner et al., 2024), employees (Van den Broeck et al., 2021), teachers (Li & Yao, 2022), and students (Howard et al., 2021). Within the context of athletes in sports clubs, findings largely mirror those observed in these domains, showing that autonomous motivation (intrinsic and identified regulation) was negatively associated with athletes’ intentions to drop out, whereas amotivation was positively associated with dropout intentions (Fabra et al., 2023; Saward et al., 2024; J. H. Yang et al., 2025). Findings for controlled motivation were, like in other contexts, less consistent: when treated as a single construct, it was positively related to dropout intentions and negatively to continuation intentions (Fabra et al., 2023; Monteiro et al., 2026). However, studies examining its subcomponents indicated that external regulation was strongly positively related to dropout intentions, while introjected regulation showed weaker or no associations (Saward et al., 2024; J. H. Yang et al., 2025). These patterns are corroborated by meta-analytic evidence: Bäck et al. (2022) found that self-determined motivation predicted continued team sport participation, while Zhang et al. (2022) identified intrinsic motivation as a medium-sized predictor of persistent sport behavior, patterns also supported by a comprehensive meta-review of SDT research across sport, education, and work (Ryan et al., 2022).

### 1.2. Beyond the Athlete: How the Coach–Athlete Relationship Shapes Sport Outcomes

The above-mentioned SDT-based research in sport has traditionally adopted an individual-centered perspective, suggesting that athletes’ participation and persistence are shaped by their own motivation (Liu et al., 2025). However, an emerging body of work emphasizes that characteristics such as motivation are co-constructed within relationships and cannot be fully understood when individuals are studied in isolation (Fonteyn et al., 2022; Campo et al., 2022). An important methodological advance in relational modeling within sport psychology has been the application of the Actor–Partner Interdependence Model (APIM; Kenny et al., 2006) to the coach–athlete relationship. APIM conceptualizes coach–athlete interactions as dyadic and bidirectionally interdependent, enabling researchers to examine both actor and partner effects within a one-with-one design (Kenny et al., 2006; Fonteyn et al., 2022). More precisely, actor effects capture how a member’s own characteristics influence their own outcomes, whereas partner effects reflect how one member’s characteristics affect the outcomes of the other member of the dyad.

APIM research on coach–athlete relationships has evolved into a small but steadily expanding body of work, examining how coaches’ and athletes’ characteristics—namely, their perceptions of their own feelings, thoughts, and behaviors—related to both their own outcomes (actor effects) and those of their dyadic partner (partner effects). Specifically, these studies have explored how characteristics such as efficacy beliefs, personality traits, attachment styles, passion, and well-being were associated with outcomes including relationship quality (Jackson & Beauchamp, 2010; Jackson et al., 2010, 2011; Davis et al., 2013; S. X. Yang et al., 2015; Jowett et al., 2013), well-being (Stebbings et al., 2016), and affect (Fonteyn et al., 2024; Denul et al., 2026).

Across these studies, robust actor effects showed that coaches’ and athletes’ own characteristics related to their own outcomes. At the same time, albeit less consistent, partner effects underscored the bidirectional influence within the coach–athlete dyad. For instance, coaches’ own confidence in the athlete’s abilities has been associated with athlete commitment and effort (Jackson & Beauchamp, 2010; Jackson et al., 2010), while coaches’ own perceptions of their supportive personality traits have shown to foster athlete closeness, satisfaction, and commitment (Jackson et al., 2011; Davis et al., 2013; S. X. Yang et al., 2015). Similarly, coaches’ pre-training well- and ill-being have been linked to changes in athletes’ well-being over the course of the training sessions (Stebbings et al., 2016). Building on these findings, Fonteyn et al. (2022) argued that actor and partner effects may also emerge for other coach and athlete characteristics, such as motivation.

### 1.3. From Dyads to Teams: Towards a Better Understanding of Sport Motivation Interdependence

Although APIM research clearly demonstrates the importance of dyadic interdependence in sport, including for motivation, an important question remains: *How do these interdependent processes unfold within team settings?* In such contexts, motivational dynamics may arise between coaches and multiple athletes, as well as also through interactions among athletes, making it essential to consider both coach–athletes and athlete–peer relationships. To study the coach–athlete relationship within team settings, we consider a one-with-many (OWM) design perspective as an extension of APIM. In this approach, one person (the coach) interacts with many others (the athletes), but the interdependence is not symmetric, as it is in traditional dyads. The OWM extension of the APIM accommodates this asymmetry while allowing the estimation of actor and partner effects. Actor effects capture whether individuals’ own motivation (coach or athlete) relates to their own intentions to discontinue involvement. For athletes, this refers to intentions to quit their sport (or drop out); for coaches, to intentions to quit coaching. In our setting, negative actor effects indicate that one’s own higher motivation is associated with their own lower intentions, which we expect to observe for more adaptive forms of motivation (i.e., intrinsic motivation and identified regulation). In contrast, positive actor effects indicate that one’s own higher motivation is associated with their own higher drop-out intentions. We expect this effect to emerge for the less adaptive forms of motivation (i.e., introjected regulation, and especially external regulation and amotivation). Partner effects, in turn, capture whether coaches’ motivation is related to their athletes’ drop-out intentions, and whether the average motivation of the athletes within the team is related to the coach’s intentions. Because the APIM-OWM diverges from the traditional dyadic APIM in this regard, we refrain from formulating a priori expectations for these effects. However, should partner effects emerge, we expect them to mirror actor effects, with negative associations for adaptive forms of motivation and positive associations for less adaptive forms.

While informative, the APIM-OWM does not capture a crucial part of interdependence in sport teams: the potential for reciprocal interdependencies within athlete–peers relationships. Addressing this gap is important because athletes’ experiences may be closely related to the (shared) motivations among peers within the team. This is consistent with the social identity approach, which posits that the social groups that athletes perceive themselves to belong to have an important impact on their behavior (Campo et al., 2022; Fransen et al., 2020; Koo et al., 2025). To capture these group-based influences, the present study applies the Group Actor–Partner Interdependence Model (GAPIM). GAPIM is used to decompose motivation among athletes within sports teams, examining its relation with dropout intentions. In addition to traditional actor effects, this model estimates several group-level effects. First, the ‘group (−1) effect’ captures the influence of the peers’ motivations on the individual athlete, showing whether participation in a team with highly motivated peers is associated with lower (negative effect) or higher (positive effect) dropout intentions. Secondly, the ‘actor–group product term’ is the product of an athlete’s own and their peers’ motivation; a negative effect therefore indicates lower dropout intentions when an athlete’s motivation corresponds to that of their peers, and higher dropout intentions when the two diverge. Third, the ‘group (−1) similarity effect’ captures the role of motivational homogeneity among peers. Because empirical evidence on these group-based effects in sport teams is still limited, these effects are examined in an exploratory manner without specific a priori hypotheses.

## 2. Materials and Methods

### 2.1. Procedure

Under the supervision of the lead authors, a group of university students initiated the recruitment process by contacting coaches of sports teams engaged in either team sports (e.g., football, basketball) or individual sports practiced in group settings (e.g., gymnastics, dance). Coaches who agreed to participate filled out a questionnaire and were also asked to forward the study invitation to a minimum of five randomly chosen team members, who did the same. In these questionnaires, both coaches and athletes reported their perceptions of motivation as well as their intentions to drop out (athletes) or quit coaching (coaches; see “Measures”). The study was conducted in accordance with the guidelines of the Ethics Committee of the Faculty of Psychology and Educational Sciences. All participants provided informed consent, confirming that they were aware of the purpose of the study, that participation was voluntary, that they could withdraw at any time without consequences, and that their responses could be used for research purposes. Participants under the age of 18 (43% of the total athlete sample) were required to obtain parental or legal guardian consent by submitting a signed informed consent form (either online or on paper). The recruitment followed a convenience sampling approach.

### 2.2. Sample

The sample comprised 51 sports teams, each representing a different club, practicing a variety of sports including football (51%), basketball (12%), gymnastics (10%), volleyball (8%), and dance (4%). In total, within these 51 teams, 268 athletes and their coach took part in the study, except for one coach, whose athletes participated but who did not complete the questionnaire. Of the participating coaches, 40 (80%) were male and 10 (20%) were female. Their mean age was 43.08 years (*SD* = 11.92; range = 19–68). Of the athletes, 187 (69.8%) were male, 80 (29.9%) were female, and 1 (0.4%) preferred not to disclose their gender. Athletes were on average 20.67 years old (*SD* = 6.84; range = 12–48). They competed at either the regional level (*N* = 176, 65.7%) or the (inter)national level (*N* = 92, 34.3%). The number of respondents per team ranged from 3 to 12, with a mean of 5.25 athletes per team. Because only a subset of athletes within each team participated, the group-level GAPIM predictors (group mean, similarity) are based on partial team coverage, which should be kept in mind when interpreting these effects. The number of training hours per week was 6.74 (*SD* = 3.78; range 1–28).

### 2.3. Measures

To maximize conceptual precision in athletes’ and coaches’ perceptions of motivation and intentions to discontinue involvement, we used instruments specifically tailored to each group, all of which have been developed and psychometrically validated in previous research. All items were rated on a 7-point Likert scale ranging from 1 (does not describe me at all) to 7 (describes me extremely well).

#### 2.3.1. Athlete Perceptions

*Intentions to drop out.* Athletes’ dropout intentions were measured using two items from the questionnaire developed by Gillet et al. (2012): ‘‘I often intend to drop out from my sports’’ and ‘‘I am determined to continue participating in my sports (reversed-scored)’’. Internal consistency, as assessed with Cronbach’s Alpha (α), was excellent: 0.85. Pearson’s correlation was 0.74.

*Motivational regulations*. Athletes’ motivational regulations were assessed using the Behavioral Regulation in Sport Questionnaire (BRSQ; Assor et al., 2009; Lonsdale et al., 2008), a valid and reliable instrument that has been widely used in previous research to measure sports motivation (e.g., De Muynck et al., 2021). The BRSQ is a self-report measure that assesses SDT’s five types of motivation, preceded by the stem “I put effort into my sport because…”: intrinsic motivation (4 items, α = 0.69; e.g., “I just like to play sports”), identified regulation (4 items, α = 0.77; e.g., “because I personally value the benefits of my sport”), introjected regulation (8 items, α = 0.80; e.g., “I have to prove myself to be a talented athlete”), external regulation (8 items, α = 0.87; e.g., “I would feel like a failure if I gave up”), and amotivation (4 items, α = 0.89, e.g., “but the reasons why I do sport are no longer clear to me these days”).

#### 2.3.2. Coach Perceptions

*Intentions to quit coaching.* Coaches’ intentions to quit coaching at the sports club were measured using a Dutch version of the turnover intention scale developed by Wayne et al. (1997; see De Clerck et al., 2024). In this study, we used two items (i.e., “I am seriously considering quitting my coaching at the sports club” and “I often think of quitting my coaching at the sports club”) that showed excellent internal consistency (α = 0.88). 

*Motivational regulations*. Coaches’ motivational regulations were measured using a Dutch questionnaire developed in previous research to assess coaches’ motivation (e.g., De Clerck et al., 2021). For this study, the stem “I put effort in my coaching because …” was used, followed by 20 items, with 4 items referring to SDT’s five motivational regulations, and more precisely intrinsic motivation (e.g., “I enjoy coaching”; α = 0.73), identified regulation (e.g., “coaching is personally important to me”; α = 0.68), introjected regulation (e.g., “I would feel guilty if I wouldn’t do so”; α = 0.70), external regulation (e.g., “because I want to give others the impression that I can perform my coaching tasks well”; α = 0.71) and amotivation (e.g., “Honestly, I don’t know why I am a coach; I really feel that I am wasting my time at the sports club”; α = 0.69).

### 2.4. Plan of Analyses

First, descriptives and correlations among the study variables were calculated. To estimate the one-with-many (OWM) extension of the Actor–Partner Interdependence Model (APIM), following the guidelines of Kenny et al. (2006), we fitted a multilevel model in which individuals (the coaches and their athletes) were nested within teams. A random intercept at the team level was included to account for nonindependence among outcomes associated with the same coach. The APIM-OWM design distinguishes two structurally different roles—the *One* (coach) and the *Many* (athletes). In the APIM-OWM, six effects were specified within each team: (a) an intercept for the coach; (b) an intercept for the athletes; (c) the actor effect of the coach (the effect of their own motivation); (d) the actor effect of the athletes (the effect of their own motivation); (e) the partner effect of the coach on their athletes (the effect of the coaches’ motivation on their athletes); and (f) the partner effect of the athletes on their coaches (the effect of average motivation of the athletes within a team on their coach).

The APIM-OWM simultaneously models the outcome of coaches (index k) and athletes (index i, k) nested within teams:

DROPOUT_coach,k_ = β_0coach_ + β_1coach_ Actor_coach,k_ + β_2coach_ Partner_athletes->coach,k_ + *u*_0k_ + ε_coach,k_

DROPOUT_i,k_ = β_0athlete_ + β_1athlete_ Actor_athlete,k_ + β_2athlete_ Partner_coach->athlete,k_ + *u*_0k_ + ε_ik_

where Actor captures one’s own predictor value and Partner captures one’s others predictor value. The random intercept *u*_0k_ captures the between-team variability, and ε_coach,k_ and ε_ik_ the residual error terms in coaches and athletes, respectively.

Next, we estimated the Group Actor–Partner Interdependence Model (GAPIM). Following the guidelines of Kenny and Garcia (2012), we used a multilevel model in which athletes were nested within teams. A random intercept at the team level was included to account for nonindependence among outcomes associated with members of the same team. In GAPIM, four predictors were specified within each team: (a) Actor: $X_{ik}$ (player *i*’s own score); (b) Group(−1): ${\bar{X}}_{-i,k}$ (mean of all *other* teammates in team *k*), (c) Actor–Group product term (hereafter referred to as the actor × peer term): $X_{ik}\times {\bar{X}}_{-i,k}$, (d) Group(−1) Similarity: average pairwise product among the others (i.e., the mean of all pairwise products among teammates excluding player). This follows the canonical GAPIM definitions and is suitable for continuous predictors.

To prepare GAPIM predictors, all continuous variables were effect-coded to range approximately between −1 and +1. This approach, adapted from Kenny and Garcia (2012), simplifies interpretation of interaction terms and aligns with GAPIM’s original logic for categorical predictors. More precisely, the minimum observed value of continuous predictors was recoded as −1 and the maximum as +1, with intermediate values scaled proportionally:

X_ik_ = 2 × (Z_ik_ − Z_min_)/(Z_max_ − Z_min_) − 1

where Z_ik_ is the raw predictor value of player i in team k, and Z_min_ and Z_max_ the minimum and maximum observed in the sample. A detailed overview of the derivations of all four GAPIM predictors and their interpretation is shown in Appendix A Table A1. Because both predictors are expressed on a common −1 to +1 metric, the product terms take their highest values when actor and peers fall at the same end of the scale and their lowest values when they fall at opposite ends. With continuous predictors these terms capture the *combination* of actor and peer levels rather than similarity in a strict distance sense (a product near zero can reflect mid-scale scores as well as a mismatch).

To estimate the effect of all four GAPIM predictors, we used a linear mixed model with random intercepts per team:

DROPOUT_ijk_ = β_0_ + β_1_(Actor)_ik_ + β_2_(Group(−1))_jk_ + β_3_(Actor×Peer)_ijk_ + β_4_(Group(−1) Sim)_jk_ + *u*_0k_ + *e*_ijk_

with *u*_0k_ the random intercept for every team k and *e*_ijk_ the error term for player i in team k. We assume both the random intercept and error term to be normally distributed. In this model, β_1_ reflects the actor effect; β_2_ the group(−1) effect; β_3_ the actor × peer effect; and β_4_ the group(−1) similarity effect.

## 3. Results

Descriptive statistics and bivariate correlations are presented in Table 1. For athletes, autonomous forms of motivation (intrinsic motivation and identified regulation) were negatively associated with dropout intentions, whereas amotivation showed a strong positive association. External regulation was positively related to dropout intentions, while introjected regulation was not significantly associated. For coaches, intrinsic motivation was negatively and amotivation positively associated with intentions to quit coaching. No significant cross-level associations were observed between coaches’ motivation and athletes’ dropout intentions or vice versa.

Table 2 presents the parameter estimates of the APIM-OWM models. No partner effects were observed; only actor effects emerged, largely consistent with expectations. For athletes, intrinsic motivation, identified regulation, and external regulation showed significant actor effects in the expected directions, whereas introjected regulation did not (see Table 2 for estimates). For coaches, significant actor effects emerged for intrinsic motivation (negative) and amotivation (positive). Overall, these findings underscore the primacy of individuals’ own motivational regulations in explaining intentions to discontinue involvement.

Table 3 reports the parameter estimates of the GAPIM. For intrinsic motivation, a significant negative actor effect was found. For identified regulation, the actor × peer product term was significantly negative, indicating that dropout intentions were higher when athletes’ identified regulation diverged from that of their peers than when the two corresponded. For amotivation, a strong positive actor effect was accompanied by a significant negative group(−1) effect and a significant negative actor × peer term: athletes reported higher dropout intentions the more amotivated they were themselves, and lower dropout intentions the more amotivated their peers were and the more their own level corresponded to that of their peers (see Table 3 for all estimates).

## 4. Discussion

The primary aim of this study was to provide an in-depth examination of the relation between athletes’ motivation and their dropout intentions in team settings. While the observed correlations were largely consistent with SDT and prior research (Fabra et al., 2023; Saward et al., 2024; J. H. Yang et al., 2025), they primarily reflect an individual-centered perspective that implicitly suggests that dropout intentions are shaped solely by athletes’ own motivational orientations. A key contribution of the present study is that it broadens this perspective by incorporating athletes’ social environments. Specifically, we examined the motivational roles of coaches and teams in relation to athletes’ dropout intentions, drawing, respectively, on an APIM-OWM and GAPIM.

### 4.1. Autonomous Motivation: The Crucial Role of Personal Enjoyment and Shared Values

For intrinsic motivation, the findings converged across APIM-OWM and GAPIM analyses, revealing a robust negative actor effect: athletes who reported greater personal enjoyment and inherent interest expressed lower dropout intentions. In both models, the social environment contributed little beyond these individual effects, suggesting that intrinsic motivation primarily operates as an individual-level resource rather than a collective one. Given that intrinsic motivation is rooted in activity-inherent enjoyment and interest (Ryan & Deci, 2024), it appears relatively less dependent on social and contextual influences.

In contrast, identified regulation emerged as a more relationally embedded form of adaptive motivation, particularly in relation to peers. While the negative actor effect in the APIM-OWM confirmed the importance of personally valuing the sport as meaningful and important (Ryan & Deci, 2024), the GAPIM results shifted the emphasis from the individual level toward the group level. Specifically, a significant negative actor × peer effect indicated that dropout intentions were higher when an athlete’s valuing of the sport diverged from that of their peers. Thus, the correspondence between an athlete’s own valuing of the activity and that of their teammates, rather than individual endorsement alone, appears relevant to dropout intentions.

One possible explanation for this finding can be found through the social identity approach, which posits that behavior of team members is not only shaped by their capacity to think, feel, and behave as individuals (i.e., personal identity) but also by their sense of themselves as group members who share values, goals, and interests with others (i.e., social identity; Fransen et al., 2015). Because identified regulation is grounded in values such as importance and meaningfulness that can be communicated, shared, and socially reinforced, teams may develop a shared understanding of what the sport is worth. Athletes whose own valuing of the sport departs from this collective understanding may therefore stand out within the team, which could render their continued participation more precarious (Fransen et al., 2015).

### 4.2. Controlled Motivation and Dropout Intentions: Constrained Effects

Contrary to autonomous motivation, the effects of controlled forms of motivation were modest. Introjected regulation showed no significant effects in either the APIM-OWM or GAPIM analyses, suggesting that self-imposed, ego-driven pressures—such as avoiding guilt or shame or enhancing feelings of pride—are not necessarily associated with elevated dropout intentions. This finding contributes to ongoing debate within the SDT literature regarding whether introjected regulation is inherently maladaptive (e.g., Van den Broeck et al., 2021). Indeed, scholars have highlighted the “Janus face” of introjected regulation, arguing that although introjected regulation may sustain engagement, such persistence may come at a cost to well-being (Saward et al., 2024; Van den Broeck et al., 2021)—an implication future research should examine more directly.

External regulation showed a significant positive actor effect in the APIM-OWM but did not reach significance in the GAPIM, suggesting that externally driven participation may function primarily as an individual-level risk factor for dropout rather than as a form of motivation related to group dynamics.

### 4.3. Amotivation: Individual Risk and the Paradox of Being Amotivated Among Amotivated Peers

With respect to amotivation, several noteworthy and theoretically challenging findings emerged. Consistent with SDT (Ryan & Deci, 2024), amotivation showed a strong positive actor effect in both APIM-OWM and GAPIM analyses, indicating that athletes who felt ineffective or disengaged were more likely to report dropout intentions. However, and perhaps more intriguingly, the GAPIM analyses also revealed significant negative group(−1) and actor × peer effects, indicating that dropout intentions were lower, for athletes at average levels of amotivation, when peers reported higher amotivation and when an athlete’s own level of amotivation corresponded more closely to that of their peers. Taken together with the positive actor effect, the model implies that dropout intentions were highest for athletes who were considerably more amotivated than their teammates. In other words, athletes’ own amotivation was clearly associated with higher dropout intentions, but this association was strongest when their amotivation stood out within the team, and weaker when teammates reported comparable levels. Teammates may thus serve as a normative reference point: where disengagement is widespread, an athlete’s own lack of motivation may be less salient as a reason to leave, whereas being noticeably more disengaged than one’s teammates may make withdrawal a more likely consideration (Fransen et al., 2015). However, these group-level amotivation findings should be interpreted with caution given their exploratory nature. An alternative explanation is that athletes whose amotivation was incongruent with their team may have already dropped out prior to data collection, leaving a more motivationally homogeneous subset. These findings should be treated as hypothesis-generating and require replication in longitudinal designs.

### 4.4. The Coach–Athlete Relationship in Team Settings: Limited Evidence for Motivational Interdependence

Finally, a notable finding of the present analyses was the absence of partner effects in the APIM-OWM models. Thus, within coach–athlete relationships in team settings, one member’s motivational regulations (i.e., the coach’s or the athletes’) were not associated with the other member’s intentions to discontinue their involvement. This finding contrasts with a substantial body of research on dyadic coach–athlete relationships, which has frequently documented reciprocal or interdependent effects (e.g., Jowett et al., 2013; Stebbings et al., 2016; S. X. Yang et al., 2015). One plausible explanation is that motivational interdependence may be particularly salient in one-to-one coaching contexts, where coaches work closely and intensively with individual athletes (Fonteyn et al., 2022). In contrast, in the team environments examined in the present study—including both team sports and individual sports practiced in group settings—coaches were responsible for multiple athletes, potentially diluting reciprocal influences.

Instead, within coach–team relationships, intentions to discontinue involvement were primarily explained by own motivational regulations, as evidenced by significant actor effects. As discussed earlier, these actor effects were evident for athletes, but importantly, they also emerged for coaches. Specifically, higher intrinsic motivation was associated with lower intentions to quit coaching, whereas amotivation was associated with higher quit intentions. In other words, coaches who experienced coaching as enjoyable and interesting were less likely to consider discontinuing their role, whereas those who felt disengaged, ineffective, or lacking a sense of purpose were more likely to consider quitting. These findings are consistent with previous research highlighting the protective role of self-determined motivation against intentions to quit coaching (e.g., De Clerck et al., 2024).

Overall, these APIM-OWM findings, together with the GAPIM findings, show that intentions to discontinue involvement in team settings are more strongly related to individuals’ own motivation than to coach–athlete crossover effects, with interdependence emerging primarily among peers.

### 4.5. Theoretical and Practical Implications

The present study offers important theoretical contributions to the literature on sport motivation by moving beyond the traditionally dominant individual-centered perspective rooted in SDT. While the strong and consistent actor effects observed across both APIM-OWM and GAPIM analyses reaffirm that athletes’ own motivation remains the primary predictor of dropout intentions, the findings also reveal that motivation is not entirely insulated from the social context. In particular, a key theoretical advancement emerging from this study is that dropout intentions depend not only on athletes’ own motivation but on how it relates to that of their teammates. Across both regulations, the peer-related effects converged on the same theme: what mattered was not only how motivated athletes were, but how their motivation compared with that of their teammates. Athletes reported the highest dropout intentions when their identified regulation was low relative to a team that valued the sport highly, and when their amotivation was high relative to teammates who were comparatively engaged. In both cases, athletes whose motivation lagged behind that of the group appeared most at risk, suggesting that teammates may function as a motivational reference point against which athletes evaluate their own intentions to continue. Because team norms, social comparison, and belonging were not assessed, this interpretation remains a hypothesis for future research rather than a mechanism supported by the present data.

From a practical standpoint, these findings suggest that interventions aimed at reducing dropout intentions should continue to prioritize fostering autonomous motivation at the individual level, particularly by enhancing intrinsic motivation and identified regulation while reducing amotivation. If the observed peer-level effects replicate, practitioners may also benefit from attending to the motivational composition of teams, for instance by being attentive to athletes whose motivation differs markedly from that of their teammates (e.g., Fransen et al., 2015). These implications remain tentative, as the data capture associations at a single point in time and do not establish that altering a team’s motivational composition would reduce actual dropout. Intervention studies with behavioral outcomes are needed to support such recommendations.

### 4.6. Limitations and Future Research Directions

Several limitations of the present study should be acknowledged, which also offer avenues for future research. First, the cross-sectional design precludes any conclusions about causal directionality. Longitudinal and experience-sampling designs would be particularly valuable in capturing the dynamic and potentially reciprocal nature of motivational processes within teams.

Second, as a group of university students initiated the recruitment process and coaches were asked to forward the questionnaires to a minimum of five randomly chosen athletes, we have no data on response rate among coaches and athletes, which may have reduced the representativeness of the sample.

Third, the study relied exclusively on self-report measures, which may be subject to common method bias and social desirability effects. Future studies could benefit from incorporating multi-method approaches, such as observational assessments of team climate, peer ratings, or physiological indicators of engagement. In addition, the measurement of dropout intentions rather than actual dropout behavior constitutes an important limitation, as intentions do not always translate into action. Future research should aim to link motivational dynamics to objective retention or dropout data.

Fourth, although the use of APIM-OWM and GAPIM represents a methodological strength, some constraints should be noted regarding the sample and design. The sample was recruited through convenience sampling, and no a priori power analysis was conducted, as the multilevel APIM-OWM and GAPIM frameworks lack established guidelines for sample size determination. Teams varied in size, the number of athletes per team was relatively modest, and only a subset of athletes within each team participated. This partial team coverage means that the GAPIM’s group-level predictors are based on incomplete information about the team’s motivational composition, which may attenuate group-level effects. Given the exploratory nature of the study and the number of statistical tests conducted, the group-level findings in particular should therefore be considered preliminary. Future studies with larger samples, more complete team coverage, and adequately powered designs could further test the robustness of the present findings.

Fifth, the study focused on coaches and peers as sources of social influence within the sports club. However, parents have also been shown to shape athletes’ motivation (De Muynck et al., 2021). Future research could clarify the relative and combined contributions of coaches, peers, and parents in shaping athletes’ motivational trajectories and dropout intentions.

Finally, given the exploratory nature of this study, no corrections for multiple testing were applied. Future studies should therefore seek to replicate our findings—especially the observed actor × peer patterns—in independent samples and consider applying appropriate multiplicity corrections to evaluate the robustness of the observed effects. Because the isolated group-level effects that reached *p* < 0.05 provide much of the basis for our claims regarding peer motivational interdependence, these effects should be regarded as preliminary, and independent replication will be needed before firm conclusions are drawn from them. Additionally, future studies could explore whether similar effects emerge for other psychological constructs, such as well-being, cohesion, or perceived competence, thereby extending the applicability of the GAPIM.

## 5. Conclusions

The present study sought to advance understanding of dropout intentions in sport teams by integrating an individual and social perspective on motivation through the combined use of APIM-OWM and GAPIM. Consistent with SDT, the findings reaffirm that athletes’ own motivational regulations—particularly intrinsic motivation, identified regulation, and amotivation—are strongly associated with intentions to discontinue sport participation. At the same time, the results provide novel insights into how motivational processes unfold within team contexts, highlighting that athletes’ experiences are not solely determined by their individual motivation but are also related to the motivational dynamics within their peer group.

## Figures and Tables

**Table 1:** Descriptives and correlations among study variables.

**Panel A: Athletes (*n* = 268)**								
Variable	*M*	*SD*	1	2	3	4	5	6
1. Intrins	6.07	0.82						
2. Identif	5.69	1.03	0.61 **					
3. Introj	3.88	1.25	0.13 *	0.20 **				
4. Extern	2.36	1.17	−0.25 **	−0.12	0.55 **			
5. Amot	1.94	1.30	−0.61 **	−0.44 **	0.16 *	0.40 **		
6. Drop	2.27	1.50	−0.52 **	−0.40 **	−0.04	0.28 **	0.66 **	
**Panel B: Coaches (*n* = 50)**								
Variable	*M*	*SD*	1	2	3	4	5	6
1. Intrins	6.47	0.81						
2. Identif	5.41	1.14	0.47 **					
3. Introj	2.35	0.94	0.16	0.26				
4. Extern	1.43	0.64	−0.34 *	−0.06	0.30 *			
5. Amot	1.20	0.67	−0.71 **	−0.29 *	0.00	0.35 *		
6. Quit	2.01	1.29	−0.31 *	−0.13	0.16	0.21	0.33 *	

Note. Intrins = intrinsic motivation; identif = identified regulation; introj = introjected regulation; extern = external regulation; amot = amotivation; drop = dropout intentions; quit = intentions to quit coaching. Cross-level correlations between athlete and coach variables were non-significant and are omitted for clarity. * *p* < 0.05, ** *p* < 0.01.

**Table 2:** Results from the full APIM-OWM model.

Predictor	Actor Athlete β	Actor Coach β	Partner Athletes ^1^ β	Partner Coach β
Intrinsic	−0.90 (0.09) ***	−0.50 (0.22) *	−0.10 (0.11)	0.05 (0.40)
Identified	−0.57 (0.08) ***	−0.15 (0.17)	0.09 (0.10)	0.17 (0.40)
Introjected	−0.05 (0.07)	0.25 (0.23)	0.15 (0.12)	−0.16 (0.30)
External	0.32 (0.07) ***	0.45 (0.31)	0.11 (0.17)	−0.17 (0.33)
Amotivation	0.76 (0.05) ***	0.62 (0.25) *	−0.01 (0.14)	0.02 (0.19)

Note. ^1^ partner effects of mean scores of athletes within the team; * *p* < 0.05, *** *p* < 0.001.

**Table 3:** Results from the full GAPIM.

Predictor	Actor β	Group(−1) β	Actor × Peer β	Group(−1) Sim β
Intrinsic	−2.11 (0.75) **	0.78 (1.42)	0.05 (1.24)	−1.42 (1.19)
Identified	0.09 (0.75)	2.29 (2.09)	−3.27 (1.40) *	−1.32 (1.46)
Introjected	−0.13 (0.22)	0.07 (0.47)	0.43 (1.04)	0.15 (0.90)
External	0.89 (0.51)	0.80 (0.83)	−0.16 (0.98)	−0.02 (0.94)
Amotivation	1.57 (0.40) ***	−1.40 (0.66) *	−1.29 (0.60) *	−0.58 (0.60)

Note. * *p* < 0.05, ** *p* < 0.01, *** *p* < 0.001.

## Data Availability

The data presented in this study are available on request from the corresponding author due to privacy reasons.

## Appendix A

**Table A1:** Computation of the GAPIM predictors at the athlete level, adapted from Kenny and Garcia (2012).

Variable	Formula	Explanation of Terms	Interpretation
1. Actor motivation	*X_ik_* = 2 ∗ [*Z_ik_* − *Z_min_*]/[*Z_max_* − *Z_min_*] − 1	– *X_ik_*: effect-coded motivation score of athlete *i* in team *k* – *Z_ik_*: the raw motivation score of athlete *i* in team *k* (e.g., their self-reported level of intrinsic motivation or amotivation) – *Z_min_* and *Z_max_*: the smallest and largest value in the sample, respectively	Positive values (up to +1) indicate that the actor scores high on the motivational regulation, negative values (up to −1) indicate that the actor scores low on that regulation compared to the values in the entire sample.
2. Group(−1) motivation	*X*′*_ik_* = [*n_k_* ∗ $\overset{-}{X}$*_k_* − *X_ik_*]/[*n_k_* − 1]	– *X*′*_ik_*: teammates’ motivation for athlete *i* in team *k* (i.e., the average motivational level of the team excluding the actor) – *n_k_*: team size of team *k* – $\overset{-}{X}$*_k_*: mean motivation in team *k*	Positive values (up to +1) indicate that the actor’s teammates on average score high on the motivational regulation, negative values (up to −1) indicate that the actor’s teammates have a low average level on that regulation.
3. Actor × peer product term	*I_ik_* = *X_ik_* ∗ *X*′*_ik_*	– *I_ik_*: actor × peer product term for athlete *i* in team *k* (i.e., product term of actor and teammates’ motivation)	Positive values (up to +1) indicate that the athlete and their teammates fall at the same end of the scale (both relatively high or both relatively low), negative values (up to −1) indicate that they fall at opposite ends of the scale.
4. Group(−1) similarity	*I*′*_ik_* = [*n_k_* ∗ $\overset{-}{X}$^2^*_k_* − *σ*^2^(*X_k_*) − 2 ∗ *I_ik_*]/[*n_k_* − 2]	– *I*′*_ik_*: teammates’ similarity for athlete *i* in team *k*, i.e., the interaction of all pairs of the others’ effects, or how similar the other *n* − 1 members of team *k* are to each other – *σ*^2^(*X_k_*): variance of the effect-coded motivation scores within team *k*	Positive values (up to +1) indicate that the actor’s teammates fall at the same end of the scale as one another, negative values (up to −1) indicate that they fall at opposite ends of the scale.

Note. All GAPIM predictors are based on effect-coded motivation scores *X*, which range from −1 to +1 and are obtained by rescaling the raw motivation scores *Z* as shown in row 1.
